# Isolated Coarctation of the Aorta: Current Concepts and Perspectives

**DOI:** 10.3389/fcvm.2022.817866

**Published:** 2022-05-25

**Authors:** Ami B. Bhatt, Maria R. Lantin-Hermoso, Curt J. Daniels, Robert Jaquiss, Benjamin John Landis, Bradley S. Marino, Rahul H. Rathod, Robert N. Vincent, Bradley B. Keller, Juan Villafane

**Affiliations:** ^1^Departments of Internal Medicine and Pediatrics and Division of Cardiology, Harvard Medical School, Boston, MA, United States; ^2^Section of Cardiology, Department of Pediatrics, Baylor College of Medicine, Houston, TX, United States; ^3^Departments of Pediatrics and Internal Medicine, The Ohio State University Medical Center, Columbus, OH, United States; ^4^Department of Cardiovascular and Thoracic Surgery and Department of Pediatrics, UT Southwestern Medical Center, Dallas, TX, United States; ^5^Department of Pediatrics and Department of Medical and Molecular Genetics, Indiana University School of Medicine, Indianapolis, IN, United States; ^6^Department of Pediatric Cardiology, Cleveland Clinic Children's, Cleveland, OH, United States; ^7^Department of Pediatrics, Harvard Medical School, Boston, MA, United States; ^8^Department of Pediatrics, New York Medical College, Valhalla, NY, United States; ^9^Cincinnati Children's Heart Institute and the Department of Pediatrics, University of Cincinnati, Cincinnati, OH, United States

**Keywords:** coarctation of the aorta, congenital heart disease, catheter intervention, heart surgery, adult congenital heart disease

## Abstract

Current management of isolated CoA, localized narrowing of the aortic arch in the absence of other congenital heart disease, is a success story with improved prenatal diagnosis, high survival and improved understanding of long-term complication. Isolated CoA has heterogenous presentations, complex etiologic mechanisms, and progressive pathophysiologic changes that influence outcome. End-to-end or extended end-to-end anastomosis are the favored surgical approaches for isolated CoA in infants and transcatheter intervention is favored for children and adults. Primary stent placement is the procedure of choice in larger children and adults. Most adults with treated isolated CoA thrive, have normal daily activities, and undergo successful childbirth. Fetal echocardiography is the cornerstone of prenatal counseling and genetic testing is recommended. Advanced 3D imaging identifies aortic complications and myocardial dysfunction and guides individualized therapies including re-intervention. Adult CHD program enrollment is recommended. Longer follow-up data are needed to determine the frequency and severity of aneurysm formation, myocardial dysfunction, and whether childhood lifestyle modifications reduce late-onset complications.

## Highlights

- Most individuals with isolated coarctation of the aorta (isolated CoA) undergo successful catheter-based or surgical therapy and live into adulthood.
- Understanding the genetic and morphogenic basis of isolated CoA, prenatal detection, and management of pregnancy related issues has improved.
- Treatment of isolated CoA associated hypertension is important to reduce aortic dilation, diastolic and systolic heart failure, and atherosclerosis risk.

## Key Points to Remember

1. **Genetics**. Familial clustering of non-syndromic isolated CoA and several classic syndromes suggests a genetic basis for isolated CoA and associated vascular disease.
2. **Prenatal care and neonatal screening**. Fetal echocardiography is the cornerstone of prenatal counseling but a definitive in utero diagnosis remains challenging. Neonatal pulse oximetry screening has improved isolated CoA detection though delayed diagnosis still occurs.
3. **CV testing**. Echocardiography, supplemented with MRI or CTA, identifies aortic complications and myocardial dysfunction. Brain MRI/CTA is useful for detecting intracranial aneurysms. Exercise testing and ambulatory BP monitoring play a role in assessing BP abnormalities.
4. **Surgery**. Coarctectomy with an end to end or an extended end to end anastomosis are the procedures of choice in neonates. The ideal intervention for larger individuals with native isolated CoA remains controversial.
5. **Interventional catheterization**. Aortic stenting is beneficial in the older child able to receive a stent expanded to adult size. Transcatheter intervention is preferred over surgery in most cases of recurrent CoA.
6. **Neurodevelopmental risk**. Neurodevelopmental abnormalities may originate in fetal life and may be influenced by postnatal events. Patients with concerning neurodevelopmental symptoms, regardless of age, may benefit from multidisciplinary evaluation.
7. **Pregnancy**. Patients with normal aortic dimensions tolerate pregnancy well. TS patients with isolated CoA have greater risk for dissection and death. Cardiopulmonary exercise testing may be useful in preconception counseling. Fetal echocardiography to detect recurrence of CHD in the offspring is recommended.
8. **Aortopathy**. Genetics, native histopathology, and complications from isolated CoA repair influence development of aortic dilation, aneurysm formation, and dissection.
9. **Comorbidities**. Systemic arterial HTN is the most common comorbidity of patients with native or repaired CoA. LV systolic and diastolic heart failure are potential long-term sequelae and complications from atherosclerosis is the leading cause of late death.
10. **Lifelong CV care**. All isolated CoA patients, whether repaired or not, benefit from lifelong CV care by a cardiologist familiar with this disease.

## Introduction

Coarctation of the aorta (CoA), a congenital narrowing of the transverse and proximal descending aortic arch, may present as an isolated defect or in association other CHD (1) and most individuals with isolated CoA now undergo successful catheter-based or surgical therapy and live into adulthood (1). As patients with isolated CoA age, natural disease progression and complications resulting from therapeutic interventions become more apparent. Isolated CoA occurring in the setting of more generalized vasculopathies and connective tissue disorders also increases the risk of adverse events following intervention. Our expanded understanding of the genetic and morphogenic basis of isolated CoA, improved prenatal detection, identification of neurodevelopmental concerns and pregnancy related issues, and continued refinement in diagnostic and therapeutic techniques continues to improve treatment success. The importance of optimizing arterial, endothelial and fluid mechanics is important to reduce the incidence of isolated CoA recurrence, progressive aortic dilation, hypertension (HTN), diastolic and systolic heart failure, and atherosclerosis. This review highlights up-to-date information with the aim of improving current practice and long-term clinical outcomes.

## Morphogenic Etiologies of Coarctation of the Aorta

Vertebrate embryonic aortic arch remodeling requires a complex and synchronized process of cell migration of multiple lineages including neural crest and vasculogeneic cells, clonal expansion, and patterned cell death resulting in the final mature mammalian left-dominant aortic arch (2–6). Errors in cell migration (7), cell differentiation, and programmed cell death as well as altered biomechanical loading conditions have been shown to impact aortic arch morphogenesis (8, 9) and correlate with finding in patients with congenital heart disease (CHD) including isolated CoA (2). The term ‘coarctation‘is derived from the Latin word ‘coarctatio' which means a drawing together to make tight and generally refers to the region between the origin of the left subclavian artery proximally and the aorta-ductus junction distally (7). CoA is relatively common (5 to 8% of all CHD), however, isolated CoA in the absence of other CHD is less frequent and occurs in one per 2,500 births (10–12). The most common CHD concurrent with CoA is bicuspid aortic valve, perimembranous ventricular septal defect, and/or posterior malalignment of the outlet septum. CoA occurs in association with more complex CHD such as hypoplastic left heart syndrome (13).

## Genetic Etiology Of Coarctation of the Aorta

Genetic syndromes and familial clustering of isolated CoA support a genetic basis for isolated CoA (14–16). The known genetic etiologies for isolated CoA are heterogeneous, pleiotropic, and complicated by variable expressivity and non-penetrance (17).

### The Role of Genetic Variants on Sex Chromosomes in CoA

Turner syndrome (TS) is caused by partial or complete loss of an X chromosome in females. Approximately 7 to 12% of girls with TS present with a CoA (18), which may occur as an isolated defect or in combination with bicuspid aortic valve (19, 20). Analysis of a TS cohort with heterogeneous, non-mosaic X chromosome abnormalities identified that loss of genes on the short arm of the X chromosome (Xp) may be responsible for the expression of isolated CoA phenotype (21). The precise genes remain undefined, but those which escape X inactivation and have homologous genes on the Y chromosome are of interest (21). Sex chromosome variants also likely contribute to non-syndromic CoA. For instance, large copy number variants (CNVs) on the X chromosome were enriched in males with isolated CoA (22). A targeted analysis of four X chromosome genes and the corresponding Y chromosome homologs identified candidate causal variants in the Y chromosome gene *TBL1Y* (23). Study of variants and epigenetic mechanisms on sex chromosomes in larger cohorts may further elucidate the etiology of isolated CoA.

### Autosomal Variants and CoA

Autosomal genetic causes of isolated CoA include CNVs and sequence variants. Isolated CoA has been reported in individuals with 1q21.1, 16p13.11, and 15q11.2 microdeletions (24–26). These and other CNVs, such as 15q26.2 deletion (27) and 21q22.3 duplication (22), support the diagnostic utility of genome wide copy number analysis in patients with CoA. Isolated CoA may result from gain or loss of dosage-sensitive genes in CNV loci or be due to altered transcription, as suggested by an overrepresentation of FOXC1 binding sites in rare CNVs of isolated CoA cases (28). Further study in larger cohorts will be required to define such genes and etiologic mechanisms. Moreover, determining the impact of CNVs on clinical outcomes will require multi-center collaboration.

Rarely, monogenic syndromic conditions including Kabuki (*KMT2D*) and Noonan (Ras-MAP kinase signaling pathway genes) syndromes are associated with isolated CoA (29–33). An arteriopathy diagnosis may impact the interventional approach, vessel response to intervention or injury, cardiovascular (CV) co-morbidity, and long-term outcome (34).

The potential monogenic causes of non-syndromic isolated CoA are not well-understood. Recent progress was made by a genome-wide association study of Icelanders, which identified increased frequency of a non-synonymous *MYH6* (p.R721W) variant in up to 20% of cases vs. 1% of controls (35). Whether this association generalizes to more ancestrally diverse populations is not known. Likely pathogenic variants in the gene *NOTCH1* have been identified in family studies that included patients with isolated CoA (36). A targeted study of *NOTCH1* identified a p.R1279H variant in 14% of CoA cases compared with 2% of controls (37). These studies suggest that variants with low penetrance may contribute to isolated CoA development and complex inheritance.

The identification of modifier genes may also be important in tailoring management strategies in isolated CoA patients. Inherited variants in the gene *MYH6* have been associated with left ventricular (LV) dysfunction in patients with isolated CoA and other left-sided defects (38). Altered aortic expression of the natriuretic gene *NPRC* may contribute to post-repair HTN in isolated CoA (39). Such studies have begun to pave the way toward using genetic testing for risk stratification clinically and identifying novel drug targets.

## Clinical CV Genetics Evaluation in Isolated CoA

The goals of a CV genetics evaluation are to: (1) identify the presence of genetic syndromes; (2) recommend genetic testing options which may include genome wide screening or targeted testing; (3) counsel families regarding the clinical implications of genetic test results; and (4) facilitate testing at-risk family members. Chromosomal microarray testing is an effective first test for many infants with severe CHD including isolated CoA (40) and may establish a syndromic diagnosis though features are subtler during infancy (41). Early involvement of genetics providers in the care of isolated CoA patients also increases the detection of silent but clinically relevant CHD in first-degree relatives as the genetic causes of CHD are increasingly recognized.

## Prenatal and Postnatal Isolated CoA Diagnosis

Detailed aortic arch assessment is part of a comprehensive fetal echocardiogram (Echo) (42). In-utero diagnosis of isolated CoA is challenging due to the presence of the patent ductus arteriosus (PDA) and some patients with isolated CoA elude early detection (43). Right ventricle enlargement, up to 1.5x the left ventricle (LV) dimensions, may be a normal finding in utero, but also occur in isolated CoA (44, 45). Significant fetal ventricular size discrepancy, great vessel disproportion, abnormal arch ratio of the isthmus diameter to the ductal diameter <0.74 (46), and Doppler abnormalities across the foramen ovale and along the aortic arch raise the suspicion for isolated CoA and warrant serial evaluation as pregnancy progresses (47–49). Investigational in utero interventions such as maternal hyperoxygenation to promote growth of the aortic arch, requires further study and risk benefit assessment (50, 51). When fetal evaluation identifies CHD and ductal-dependent systemic blood flow, delivery at an institution capable of providing prostaglandin E1 and post-natal CV management is recommended.

Isolated CoA may be detected by neonatal pulse oximetry screening for critical CHD due to right to left ductal shunting and lower oxygen saturations in the lower extremities (52–54). The peripheral perfusion index, a derived pulse oximetry parameter, and photoplethysmography are promising non-invasive indicators with the potential to improve isolated CoA detection (55, 56). Despite neonatal screening, a delayed diagnosis of isolated CoA may still occur (57) resulting in infants present in cardiogenic shock and LV dysfunction.

**Figure 1:**
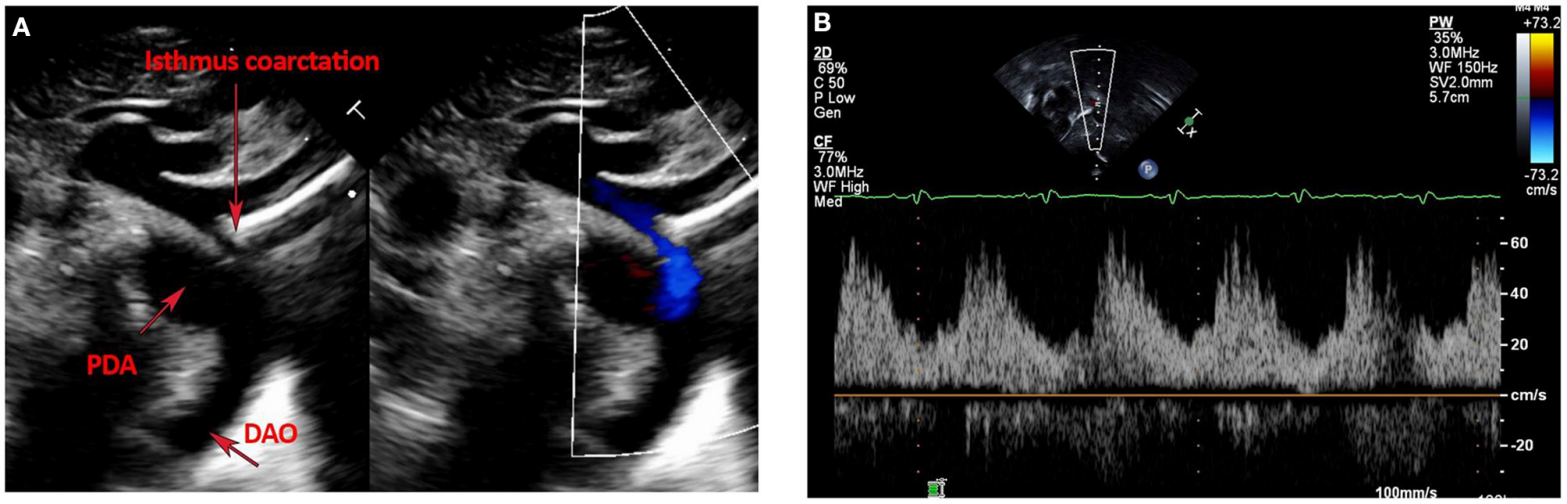
**(A,B)** Echocardiographic images of Isolated CoA. **(A)** Shows B-Mode and color Doppler images of discrete isthmus CoA with obvious size discrepancy between the isthmus and PDA diameters. **(B)** Shows an abnormal abdominal aorta Doppler profile with a low velocity signal, blunted upstroke with delayed systolic peaking, a slurred downstroke and continuous diastolic flow in a patient with isolated CoA.

Postnatal echo is the mainstay in diagnosing isolated CoA and associated CHD lesions, in defining the presence, size, and direction of ductal shunting, and in measuring the ascending aorta, transverse arch, isthmus, CoA length and descending aorta dimensions. Echo is safe, readily available, and cost-effective. The suprasternal notch view provides visualization of areas of isolated CoA and allows Doppler gradient measurements (Figure 1A). Delayed systolic peaking with diastolic runoff in the abdominal aortic Doppler flow profile suggests the presence of arch obstruction (Figure 1B).

The carotid-subclavian index, defined as the distal transverse arch diameter divided by the distance between the left common carotid and left subclavian arteries, may aid in identifying neonates at risk for isolated CoA even when a PDA may mask its presence (49).

Neonatal isolated CoA has a spectrum of severity, ranging from asymptomatic patients with borderline small arch dimensions and a PDA to unequivocal critical arch obstruction (49). When ductal dependence is uncertain, neonates may be observed without prostaglandin E1 to allow PDA closure while undergoing active surveillance for evidence of arch obstruction. Upper and lower extremity blood pressure assessment and interval echo evaluation of Doppler gradients proximal and distal to area in question are performed until the PDA closes. This practice identifies neonates with hemodynamically significant arch narrowing requiring intervention. Neonates with isolated CoA and arch narrowing that does not reach the threshold for intervention require serial clinical reassessment over the first year of life as arch obstruction may increase during involution of remaining ductal tissue.

Isolated CoA can also present during childhood, and less commonly in adults in the absence of other CHD. Later presentation of CoA can be associated with heart failure symptoms, refractory systolic hypertension, murmur, decreased femoral pulses, and LVH on ECG (58).

**Figure 2:**
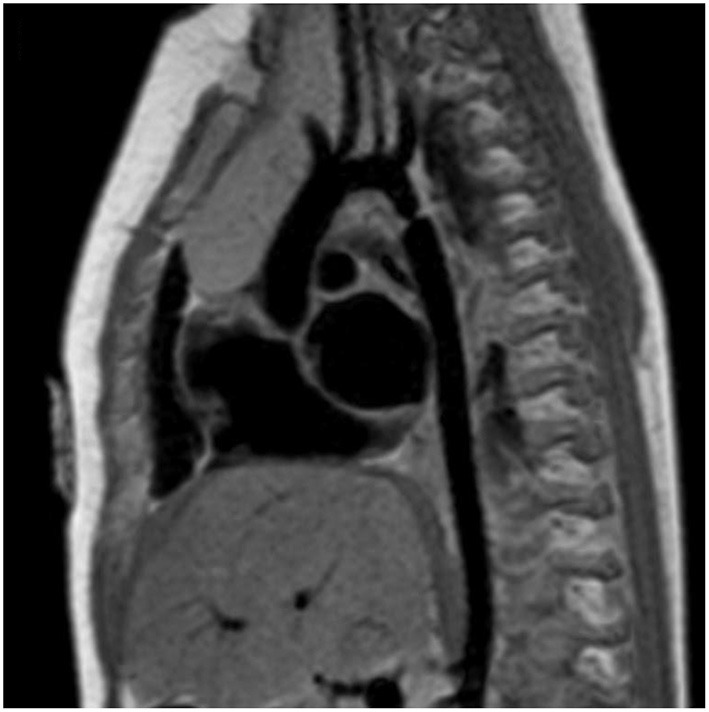
Cardiac magnetic resonance imaging (MRI) of isolated CoA. Cardiac MRI showing a discrete and isolated CoA. This modality offers high resolution imaging of the entire aortic arch, helping localize the extent and significance of the coarctation.

**Figure 3:**
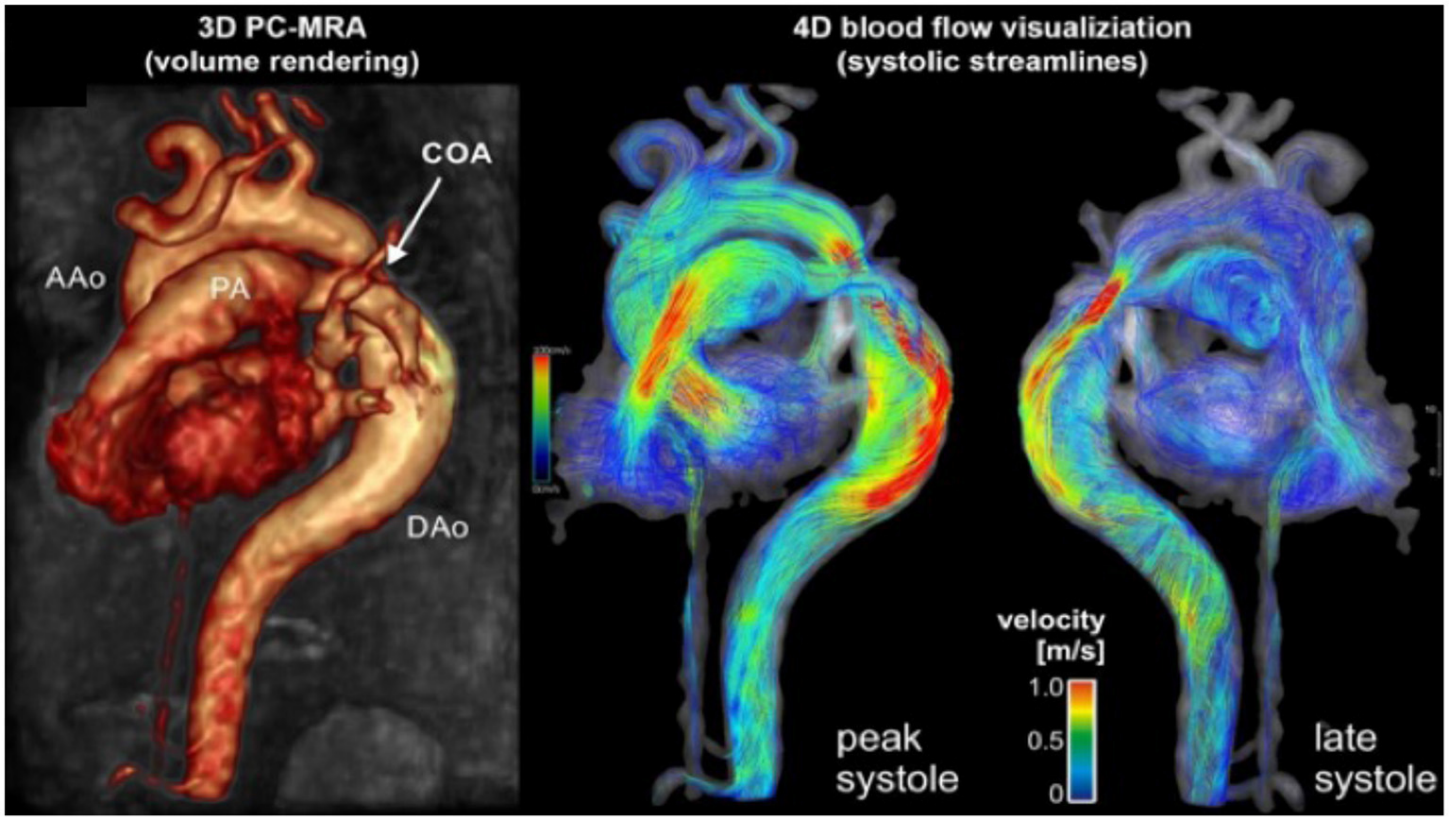
Cardiac magnetic resonance angiography (MRA) of isolated CoA with 4D flow. Advanced phase contrast magnetic resonance angiograms (PC-MRA) allows for both 3-dimensional anatomic evaluation of arch anatomy as well as 4-dimensional blood flow visualization using time resolved streamlines. Image courtesy of Joshua Robinson and Michael Markl (Dept. of Radiology, Northwestern University).

MRI and CTA complement echo with more complete 3D visualization of the thoracic aorta (Figure 2). A comparison of the strengths and weaknesses of echocardiography, MRI, and CTA are outlined in Table 1 (59–61). MRI flow analysis findings indicating increasing isolated CoA severity include decreased acceleration rate and peak and time averaged flow, delayed onset of descending aorta flow compared to the ascending aorta, prolonged deceleration with increased antegrade diastolic flow and increased descending aorta collateral flow (62). Advances in MRI 3D flow analysis (4-dimensional flow) correlate with invasively measured pressure gradients (63) (Figure 3). Future work in MRI 4D flow and computational fluid dynamics may identify patients at higher risk for developing re-coarctation and/or aneurysms (64, 65).

**Table 1:** Comparison of echocardiography, cardiac magnetic resonance imaging, and computed tomography.

	**Echocardiography**	**MRI**	**CT**
Intracardiac anatomy	Excellent structural and functional assessment, especially in younger children	Excellent structural and functional assessment	Gated cine CTs are associated with higher radiation exposure
Vascular anatomy	Limited, can miss distal obstruction and/or aneurysm formation	Excellent	Excellent, including assessment in in-stent stenosis
Technical limitations	Acoustic windows tend to worsen with age	Ferromagnetic objects result in artifacts; pacemakers are a relative contraindication	Streak artifact from contrast
Radiation exposure	None	None	Yes
Need for sedation	Generally not required in patients >3 years	Often required in patients <7 years old	No
Assessment of restenosis	Can miss distal obstructions	Excellent anatomic visualization + flow and collateral assessment	Excellent anatomic visualization but limited functional assessment
Assessment of aneurysm formation	Can miss distal or small aneurysms	Excellent anatomic visualization	Excellent anatomic visualization

## Neonatal Surgical Management of Isolated Coarctation of the Aorta

In contemporary practice, in the absence of significant aortic arch hypoplasia isolated CoA in the neonate is most commonly treated via left thoracotomy with resection (“coarctectomy”) and either end-to-end anastomosis or extended end-to-end anastomosis. The initial approach, described in the first report of successful isolated CoA repair in 1945 (66) was associated with high anastomotic stenosis rates in the smallest children. As a potential solution, modified repairs enlarged the isolated CoA with a patch employing either prosthetic material (67) or the adjacent subclavian artery (68). However, the rate of recurrent isolated CoA was not demonstrably lower with either patch technique and late aneurysms and pseudoaneurysm occurred with this approach (69). In addition, the subclavian flap procedure deprives the left arm of antegrade blood flow, though this is generally well-tolerated (70). A modification of the end-to-end anastomosis was described in 1977 where the proximal and distal segments are spatulated and “overlapped,” a procedure known the extended end-to-end anastomosis (71). Several large series have demonstrated excellent outcomes with extended end-to-end anastomosis, even in very small children, with recurrent isolated CoA rates <10% (72–75).

When the entire arch is hypoplastic, isolated CoA repair via thoracotomy may leave important residual proximal obstruction. In such cases, repair of both the arch and isolated CoA may be accomplished via sternotomy with adjunctive cardiopulmonary bypass (employing either regional low flow perfusion or deep hypothermic circulatory arrest). This may take the form of ligation of the aortic isthmus, coarctectomy, and end-to-side anastomosis of the descending aorta to the ascending aorta/proximal arch, termed “arch advancement” (76). Alternatively, the entire arch may be augmented with an allograft patch, with or without coarctectomy (77, 78).

For an isolated CoA patient with an “indeterminate” degree of arch hypoplasia, the decision about whether to address the arch is complicated by the observation that moderate hypoplasia may evolve, favorably or unfavorably. In the fetus with isolated CoA, an intact ventricular septum, and “borderline” hypoplastic LV chamber dimensions, (Z score −2 to −4) the evolution of the obstruction may be challenging to predict as LV growth may or may not ultimately support biventricular physiology. Isolated CoA associated with borderline LV is part of the spectrum of presentation of fetal CoA. Reduced LV dimensions and mass may represent a primary myocardial disorder this is separate from the etiology of coarctation. In a small series, most fetuses achieved a biventricular circulation, though a minority developed hypoplastic left heart syndrome (79). Similarly, in a cohort of 51 with neonatal mitral and aortic valve annulus Z-scores of −2 to −4, 90% had normalized valve scores at intermediate (6.1 ± 1.6 years) and long-term (15.4 ± 1.5 years) follow-up (80). There is some evidence that even a moderate to severely hypoplastic proximal arch (up to Z scores of −6) may undergo additional growth after repair of the distal isolated CoA segment with a relatively low incidence of re-intervention (81). However, despite relief of arch obstruction and adequate LV growth, some patients have prolonged hospitalization due to persistent LV diastolic dysfunction and secondary pulmonary HTN (82). In cases where there is significant pulmonary overcirculation due to an atrial level shunt after isolated CoA repair, closing the atrial septum may encourage antegrade flow through the left heart and LV growth (83). However, inadequate relief of proximal arch obstruction is associated with worse late outcomes, including persistent increased LV afterload, LV hypertrophy, and both pulmonary and systemic HTN (77). Thus, the decision to repair isolated CoA with sternotomy and CPB vs. thoracotomy without CPB remains difficult and must be based on associated intracardiac pathology, as well as local experience and results.

Though surgical repair is the preferred approach in small children with isolated CoA, temporizing palliation may be helpful in patients deemed at high surgical risk. Neonates considered too small or frail for surgical repair, typically those weighing <1.5 kg, may receive intravenous prostaglandin E1 to maintain ductal patency while awaiting somatic growth. Some high-risk patients with isolated CoA may be candidates for temporizing angioplasty and stenting (84). In such cases, subsequent surgical repair will be necessary because the small size of the initial stent will preclude dilatation to “adult” size. The very low birth weight infant with isolated CoA is particularly challenging and mortality in infants under 750 grams with CHD can exceed 65% (85, 86).

## Catheter-Based Intervention

Whereas, surgical management is preferred in the neonate due to lower risk of recoarctation and/or aneurysm formation, isolated CoA presenting later in childhood and adulthood is often amenable to percutaneous intervention. Following the first reports of balloon angioplasty for isolated CoA in the early 1980's (87), indications for catheter intervention have expanded to include most infants with native CoA, patients with recurrent isolated CoA and a transcatheter systolic gradient of >20 mmHg with suitable anatomy, and isolated CoA associated with systemic HTN (88–90). Angioplasty at any age may be considered to stabilize a critically ill patient. The decision to intervene is generally determined by non-invasive gradient measurements including BP gradient between upper and lower extremities, as well as Echo and/or Magnetic Resonance Imaging (MRI) or Computed Tomography Angiography (CTA) findings. The first multi-center report on native isolated CoA from the Valvuloplasty and Angioplasty of Congenital Anomalies (VACA) Registry confirmed that native isolated CoA could be effectively dilated in both infants and older children, with an overall complication rate of 17%, including 1 death and 8 patients who developed aneurysms (91, 92). Although dilation of native isolated CoA can be performed effectively and is relatively safe, its use remains controversial in early infancy.

When compared to angioplasty alone, balloon expandable stent implantation (SI) is thought to achieve better results. However, a weight or age at which patients become candidates for stent placement has not yet been established and small diameter stents require redilation as subjects grow to adult size. In a multicenter prospective study comparing angioplasty, SI, and surgery, the age and weight range of patients who had undergone stent placement for isolated CoA were 16.6 ± 10.9 years and 55 ± 24 kgs., respectively, the youngest being 2.2 years of age (93). Unplanned re-interventions occurred at similar rates across all groups. Additional studies have confirmed the effectiveness of coarctation stenting (94).

Data from the IMproving Pediatric and Adult Congenital Treatments (IMPACT®) registry reported on balloon angioplasty and/or stent therapy for both native and recurrent CoA in 671 patients (95). Stent implantation was utilized more commonly in older children and adults, and resulted in improved post-procedure mean systolic gradients than angioplasty alone, which was performed more commonly in infants. The Congenital CV Interventional Study Consortium (CCISC) evaluated 350 patients with native CoA treated with stenting, angioplasty, or surgery (96). Stent implantation was performed only in individuals over 2 years of age. All 3 treatment groups demonstrated significant hemodynamic improvement, although surgery and stent placement were superior in short-term follow-up. Shorter length of stay and fewer complications were noted with transcatheter procedures. Both Cheatham-Platinum^TM^ bare metal and covered stents were equally effective in relieving CoA (96, 97) though there was a slightly higher rate of post-stent aortic wall injury in the covered stent group (97). COAST II (Covered Cheatham-Platinum^TM^ Stents for Prevention or Treatment of Aortic Wall Injury Associated with isolated CoA Trial) studied the use of covered stents for both prevention and treatment of aortic wall injury, recognizing that sheath size and the potential for vascular injury may limit its use in smaller patients (96, 97). COAST II showed that Covered Cheatham-Platinum^TM^ SI covered aortic wall injury in 92% of cases with no new aortic wall injury identified (96, 97). Covered stents are preferred over bare metal stents for treatment of high-risk CoA, such those with near or acquired atresia at the CoA site (98).

The treatment of isolated CoA first recognized in adulthood requires additional consideration for concurrent disease that can affect aortic wall integrity, such as HTN and/or atherosclerosis, as well as other vascular risk factors including smoking, obesity, and diabetes (98). Treatment of native isolated CoA in the adult patient may utilize balloon-expandable covered stents or self-expanding covered stents (86, 99). In contrast to the initial treatment of native CoA, balloon angioplasty rather than SI is the usual interventional treatment of recurrent isolated CoA following initial angioplasty or surgical intervention due to the low risk of aneurysm or dissection (100) while a new stent is indicated in the setting of stent fracture. Recurrent isolated CoA can occur due to neointimal hyperplasia, stent fracture, or somatic growth, necessitating longitudinal follow-up with yearly clinical evaluations and periodic imaging (98, 99). The current published recommendations for balloon angioplasty for native and recurrent isolated CoA are summarized in Table 2 (98–101). While catheter intervention is preferred over surgery for recurrent CoA, the primary intervention is dependent on the age and size of the patient, consideration for disease recurrence with consequent re-intervention, and the center's interventional and surgical experience.

**Table 2:** Current published recommendations for balloon angioplasty: native and recurrent CoA.

**Indication**	**Class**	**Level of Evidence**
Balloon angioplasty of re-coarctation is indicated when associated with a transcatheter systolic C coarctation gradient of >20 mmHg and suitable anatomy, irrespective of patient age	I	C
Balloon angioplasty of re-coarctation is indicated when associated with a transcatheter systolic C coarctation gradient of <20 mmHg and in the presence of significant collateral vessels and suitable angiographic anatomy, irrespective of patient age, as well as in patients with a univentricular heart or with significant ventricular dysfunction	I	C
It is reasonable to consider balloon angioplasty of native CoA as a palliative measure to stabilize IIa C a patient, irrespective of age, when extenuating circumstances are present such as severely depressed ventricular function, severe mitral regurgitation, low cardiac output, or systemic disease adversely affected by the cardiac condition	IIa	C
Balloon angioplasty of native CoA may be reasonable in patients beyond 4 to 6 months of age when IIb C associated with a transcatheter systolic coarctation gradient of >20 mmHg and suitable anatomy	IIb	C
Balloon angioplasty of native or recurrent CoA might be considered in patients with complex	IIb	C
CoA anatomy or systemic conditions such as connective tissue disease or Turner Syndrome but should be scrutinized on a case-by-case basis		
Reproduced/adapted with permission from JACC (98, 100) and the American Heart Association (101).		

## Assessment and Management of Arterial Collaterals

**Figure 4:**
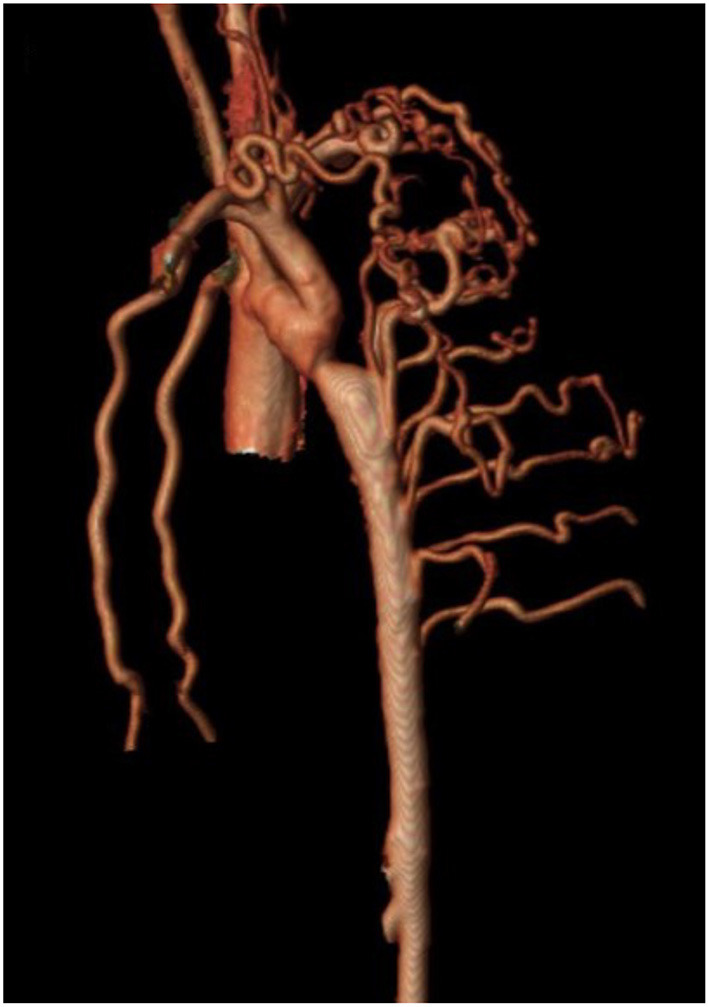
Cardiac magnetic resonance angiography (MRA) demonstrating isolated CoA with prominent collateralization. A patient with isolated CoA and prominent collateral vessel formation.

Isolated CoA is often associated with a range of arterial collateral vessels which preserve distal flow. These collateral vessels tend to be more numerous and prominent when CoA diagnosis is made at an older age. MRI analysis can quantitate collateral flow by comparing the flow at the level of the diaphragm to that in the proximal aorta (102–105). Increased collateral flow suggests more severe obstruction and may be more sensitive in assessing severity of recurrent isolated CoA when compared to measured upper and lower extremity BP gradients (65). In those with delayed presentation of native CoA, increased collateral flow is thought to diminish the risk of spinal cord ischemic injury during surgery (Figure 4). In patients with minimal collateral flow, the surgeon may choose to perform CPB or regional perfusion to decrease this risk. MRI analysis can quantitate collateral flow by comparing the flow at the level of the diaphragm to that in the proximal aorta.

## Neurodevelopmental Outcomes in Patients With Coarctation of the Aorta

Structural CHD, including isolated CoA, is associated with adverse neurodevelopmental outcome and the risk of neurodevelopmental issues is higher in more complex CHD associated with genetic syndromes and altered neurodevelopment than in isolated CHD lesions such as isolated CoA. In 2012, the American Heart Association and American Academy of Pediatrics published the first comprehensive scientific statement on the evaluation and management of neurodevelopmental issues in CHD survivors (106). This statement formally identified and stratified CHD survivors for risk of worse neurodevelopmental outcome (Table 3), outlined a surveillance, screening, evaluation and management algorithm for CHD survivors, with recommendations to optimize neurodevelopmental outcomes in this population (106). Patients at high-risk have a distinctive pattern of neurodevelopmental and behavioral impairment across a broad range of domains, including mild cognitive impairment, deficiencies in academic achievement in math and reading, deficits in social cognition, core communication skills and pragmatic language, inattention, hyperactivity and impulsivity, deficits in visual construction and perception, impaired executive functioning (organization, planning, and task-management), and limitations in gross and fine motor skills. In addition, a disproportionate number of these patients have significant behavioral or emotional issues, including Attention Deficit Hyperactivity Disorder, post-traumatic stress symptomatology, anxiety, and depression in both the patient and their parents (106). Interestingly, referral rates for neurodevelopmental assessment may be biased by providers and by anatomic diagnosis (107).

**Table 3:** Risk factors for neurodevelopmental delay in patients with CoA^*^.

Prematurity
Developmental delay recognized in infancy
Suspected genetic abnormality or syndrome associated with developmental delay
History of mechanical support or extracorporeal membrane oxygenation
Perioperative seizures
Cardiopulmonary resuscitation
Hospital stay >2 weeks
Abnormalities in neuroimaging
Other conditions determined by the health care team

* *Adapted from Marino et al. (106). Neurodevelopmental outcomes in children with congenital heart disease: evaluation and management a scientific statement from the American Heart Association. Circulation 126.9 (2012): 1143–72*.

The origins of postnatal neurodevelopmental abnormalities in patients with CHD, including CoA, likely originate during fetal life and CHD is clearly associated with a significant (28%) prevalence of structural brain abnormalities (108). For example, cellular migration events associated with abnormal neural crest migration in 22q11 deletion are well-recognized to affect the brain, heart, and vasculature, along with the face, parathyroid, and thymus. Altered cerebral blood flow correlating with head growth is seen more frequently in left heart associated CHD and in isolated CoA with aortic arch flow reversal, but not in those with antegrade flow (109). To minimize the risk of brain injury in the neonate or infant with CoA, every effort must be made to optimize systemic oxygen delivery during the perioperative period. Neonates or infants with isolated CoA who undergo a lateral thoracotomy without CPB are considered low-risk for a long-term neurodevelopmental disorder, disability or developmental delay, with the exception of those children with isolated CoA with one or more of the comorbidities listed in Table 3. Prematurity or genetic disorders or syndromes are the most common comorbidities that would identify a patient with isolated CoA as high-risk for neurodevelopmental disorder, disability, or developmental delay. Infants with isolated CoA deemed high-risk for neurodevelopmental abnormalities benefit from formal periodic developmental and medical evaluation, coordinated through their medical home. Current clinical management of children at high-risk for neurodevelopmental disorder, disability, or developmental delay includes: (1) Early referral for evaluation and intervention; even before a neurodevelopmental abnormality is recognized; (2) Periodic re-evaluation for neurodevelopmental disorders and delays at 12–24 months, 3–5 years, and 11–12 years of age; and (3) Counseling regarding educational or vocational options when patients reach young adulthood. In adults, a history of CoA repair is associated with a modest (1.4 Odds Ratio) increased risk of early onset dementia (110).

## Post-Intervention Ongoing Care and Surveillance

All patients with CoA, whether repaired or not, benefit from lifelong CV care by cardiologists with expertise in this disease (111, 112), including surveillance for post-repair aortic aneurysms which may be more common following catheter intervention than surgery (113). Beginning in early adolescence, information regarding their diagnosis, medical history, care plan and the need for surveillance and prevention strategies should be discussed (114). Healthy lifestyle choices, physical activity, health record maintenance, compliance with follow-up appointments, safe and effective contraception methods, nutrition, medications, and optimal dental care are topics of discussion (115, 116). Unfortunately, after childhood isolated CoA repair, many adult patients do not receive optimal long-term CV care. Nearly half of adult isolated CoA patients are lost to regular cardiology follow-up as they often feel well, lack adequate health insurance, and/or are unaware of potential long-term complications (116). Some patients with repaired isolated CoA incorrectly believe that they are “fixed.” Individuals lost to care have a higher incidence of complications and a greater likelihood of requiring an intervention (116).

**Figure 5:**
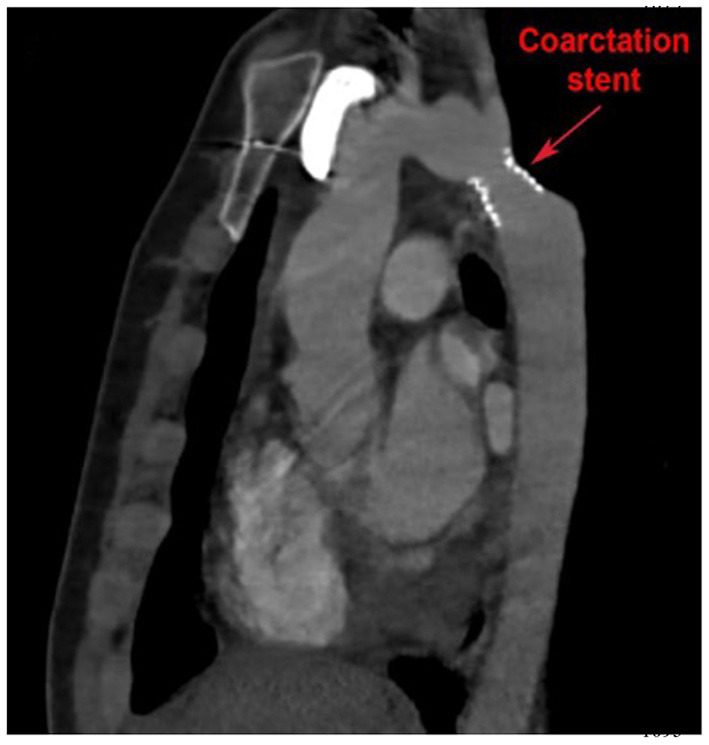
Cardiac computed tomography (CT) imaging of isolated CoA following stent implantation. Cardiac CT also allows for high resolution imaging of the entire aortic arch and enables visualization of possible in-stent stenosis.

Longitudinal surveillance of isolated CoA includes regular physical examination and interval imaging of the aorta for recurrent CoA. CTA offers a high-resolution 3D assessment of the thoracic aorta. It can be safely utilized in patients with pacemakers and is generally less sensitive to artifact from implanted devices. CTA can be used to evaluate “in-stent” stenosis in patients who have undergone percutaneous stent implantation to treat isolated CoA (Figure 5). Exposure to ionizing radiation is limitation of CTA, although technological advancements have resulted in significant radiation dose reduction, and functional assessment to quantify blood flows and velocities is limited in CTA.

It is recommended that adults with a history of isolated CoA repair should undergo 3D thoracic imaging by MRI or CTA at least every 5 years. MRI and CT are also useful as surveillance for intracranial aneurysms which can be present in 10–13% of adult patients (62–65, 117).

## Clinical Sequelae: Hypertension

Over 30% of children and more than two-thirds of adults with a history of isolated CoA repair have systemic HTN on late follow-up and this number is higher if 24-h BP monitoring is performed (118). In adults with a history of isolated CoA repair, 25–80% have HTN, affected by factors including patient age and whether BP was measured at rest, with exercise, or during ambulatory BP monitoring (119–122).

Risk factors for HTN include the use of subclavian flap or patch/graft material (123) which result in residual lesions and older age at repair (>20 years of age) (111, 124). Proposed mechanisms for persistent HTN include baroreceptor dysfunction, abnormal post-repair aortic arch morphology and impaired vascular distensibility (125, 126). Additional factors which may contribute to HTN include the presence of a cervical aortic arch, renal abnormalities, and vascular dysfunction (125). Assessment for recoarctation is essential for any patient presenting for HTN evaluation with a history of isolated CoA repair. Patients who have isolated CoA repaired during early infancy may have lower rates of HTN. In patients with HTN and significant CoA, relief of obstruction should be considered prior to medical treatment, though chronic HTN may develop despite successful transcatheter isolated CoA stent therapy. HTN is often noted in children and adolescents as early as ages 7–16 years, even in the absence of residual aortic arch obstruction.

Patients after isolated CoA repair benefit from the evaluation for exercise-related HTN as it may be a harbinger for the development of late resting HTN (127–131) and exercise testing aids in detecting any pathologic elevation in BP, even in those postoperative isolated CoA patients thought to be “normotensive” (130, 131). While exercise testing has been historically used to assess patients with a history of isolated CoA repair, the increased availability of 24-h ambulatory BP monitoring has become the preferred method to evaluate, detect, and manage HTN in children (132) and allows for detection of nocturnal HTN and exercise-induced HTN despite normal daytime BP. Adult patients with repaired isolated CoA and HTN also benefit from a home BP monitor and periodic ambulatory BP monitoring (129).

In the absence of recurrent or residual CoA, clinical management of HTN includes increased physical activity, dietary modification, weight management, and pharmacotherapy. Angiotensin converting enzyme inhibitors and beta blockers are effective in reducing BP, and enalapril has been shown to reduce LV mass index (11).

## Recoarctation and Aneurysm Formation in Adults

**Figure 6:**
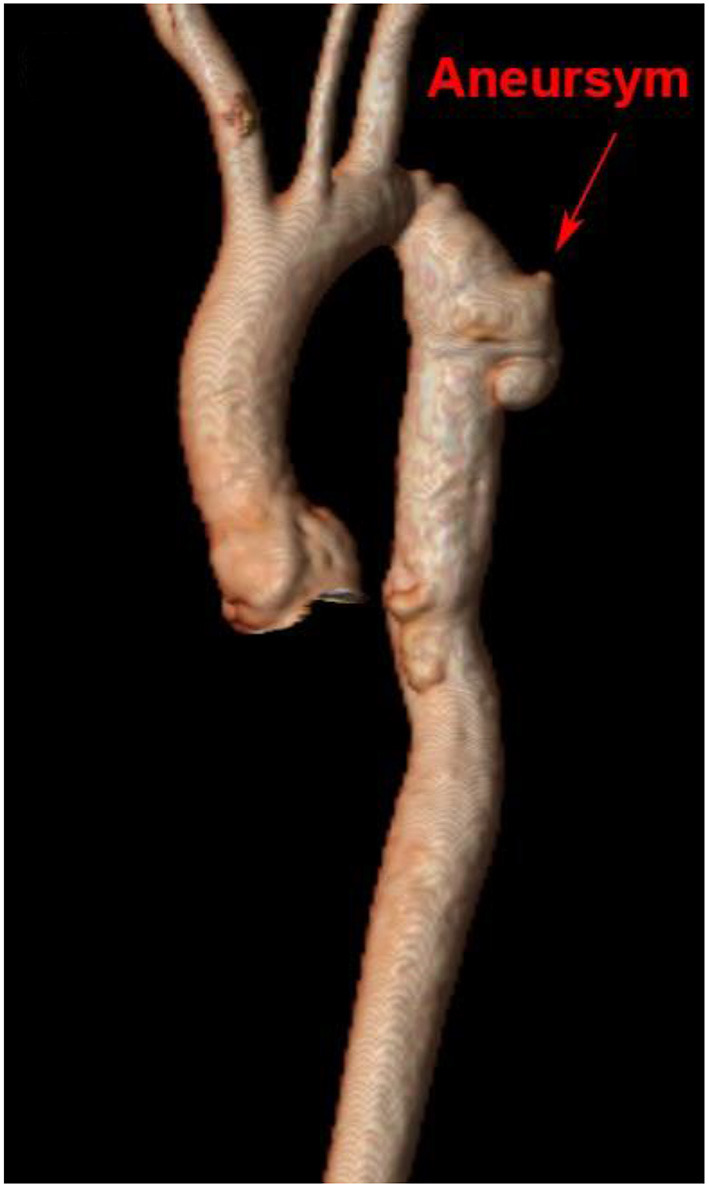
Cardiac magnetic resonance imaging after isolated CoA repair with aneurysm formation. 3D reconstruction of a patient's aorta with a Gothic arch and aneurysm formation at the site of prior isolated CoA repair.

Aortic aneurysm following CoA remains a life-long risk due to variations in vascular post-repair response that may impact growth and remodeling. Clinically significant recoarctation and/or aneurysmal dilation, diagnosed by MRI or CTA, occurs in nearly 10 and 13% of adult patients, respectively [(103), Figure 6]. Restenosis is more likely to occur after conventional resection with end-to-end anastomosis, while aneurysm formation is more frequent following surgical patch and older age at repair (132). Use of Dacron patch material is associated with a higher incidence of aneurysm formation (19–42%), morbidity, and mortality (133, 134). Reintervention for aneurysms distal to the isolated CoA repair may be performed surgically or by transcatheter approach, depending on the anatomy with a high rate of success. Fortunately, dissection following isolated CoA repair remains rare (135) and may be due to intrinsic abnormalities in the vascular wall rather than hemodynamic sequelae.

## Heart Failure in Adulthood

Individuals with repaired isolated CoA may develop LV systolic and/or diastolic myocardial dysfunction with age, which may not correlate with severity of isolated CoA or intervention history. Some individuals may have inherent cardiomyopathy with late presentation or may develop myocardial disease from the hemodynamic effects from chronic or recurrent increased afterload and abnormalities of vascular ventricular coupling (136) as defined abnormal myocardial contractility at rest and in response to exercise, with lower LV global longitudinal strain by speckle tracking echocardiography (136) and by MRI-based myocardial deformation indices.

Patients after isolated CoA repair, especially those repaired at an older age, have impaired bio-elastic properties of the thoracic aorta that may contribute to LV dysfunction (137, 138) and residual isolated CoA can be associated with LV dysfunction, even in the absence of HTN or a significant pressure gradient (139, 140).

## Aortopathy

Aortopathy in isolated CoA may contribute to aortic dilation, aneurysm formation, dissection, and death and may be asymptomatic (140). In a large series, nearly 60% of patients with repaired isolated CoA had significant aortic aneurysms, most commonly at the site of repair. Other affected areas include the ascending aorta and left subclavian artery, with multiple aneurysms seen in nearly half of the patients suggesting isolated CoA can be one component of a diffuse vasculopathy (141, 142). Isometric exercise is discouraged in cases of aortic dilation, especially in the presence of under controlled resting or exercise induced HTN.

For many of the syndromes associated with CoA, a diffuse arteriopathy causing arterial stenosis and/or aneurysm formation may predispose patients to dissection, rupture, end-organ ischemia, and procedure-related vascular complications. Cystic medial necrosis in neonates with isolated CoA suggests a congenital aortopathy, which may be influenced by the type of repair and age at intervention. Dilation of the aortic root and the mid-ascending aorta, as measured by MRI, is seen more frequently in those who also have a bicuspid aortic valve, regardless of the presence of HTN (141). Significant aortic arch abnormalities, including recurrent isolated CoA and aortic aneurysm can be detected by MRI despite the absence of symptoms, or findings on clinical exam or echo (141). Currently, no pharmacologic therapy has been proven to prevent or treat the aortopathy associated with CoA.

## Atherosclerotic CV Disease

Atherosclerotic CV disease is the leading cause of late death in patients after isolated CoA repair, with an increased prevalence of acute myocardial infarction and cerebrovascular accidents (143, 144). To our knowledge, there is no literature that identifies increased atherosclerotic risk in patients with isolated CoA beyond the secondary adverse effects of hypertension. It is unclear whether these complications are related to a primary vascular abnormality such as impaired flow-mediated vasodilation, increased levels of inflammatory cytokines, and increased carotid intima-media thickness (144). While it has been hypothesized that impairment in vascular reactivity predisposes to early atherosclerotic CV disease, isolated CoA alone does not always predict the development of coronary artery disease (145). Older age, male sex, HTN, diabetes, and hyperlipidemia increase the risk of coronary artery disease in CHD including CoA. Statin therapy has been found to improve the levels of inflammatory markers but had no effect in carotid intimal-media thickness (146, 147). It is important to identify modifiable CV risk factors and practice guideline-based management (100, 147). Further investigation is necessary to determine whether improvement in CV risk factors with or without empiric aspirin or statin therapy would prevent or retard the progress of atherosclerotic CV disease in patients with repaired CoA.

## Pregnancy

Pregnancy is well-tolerated in most patients after successful isolated CoA repair, but a comprehensive preconception evaluation including advanced imaging is recommended (148–150). Intervention prior to pregnancy may be performed to address a significant residual CoA. HTN during pregnancy is common, even in the absence of residual isolated CoA gradient, with an increased risk for preeclampsia, resulting in placental hypoperfusion, fetal growth retardation, and premature delivery (149).

Cardiopulmonary exercise testing may be useful in preconception counseling (150). It is not clear if exercise-induced HTN correlates with adverse maternal or fetal outcome. Therapy with β-blockers in selected patients with exercise-induced HTN but no residual isolated CoA may be assumed to reduce the risk of complications during pregnancy, however there is currently insufficient published data on this topic. HTN at rest without residual isolated CoA is managed with standard antihypertensive therapy, but angiotensin-converting enzyme inhibitors and angiotensin receptor-blockers should be avoided due to potential teratogenic effects.

Oocyte donation has enabled women with TS and ovarian insufficiency to become pregnant, albeit with a significant incidence of morbidity and mortality due to aortic dissection (148–152). TS women with and without a history of CoA repair are at increased risk for aortic dissection and death. Residual gradients and evidence for aortic dilation proximal or distal to the site of CoA repair can be reasonably assumed to increase aortic dissection risk. All individuals with TS should be informed about the increased risk of aortic complications, including the risk of aortic dissection and death, regardless of aortic size (151, 152). In individuals with short stature, aortic dimensions must be corrected for body surface area, as general adult reference ranges may not apply.

The pregnant patient with a history of isolated CoA is best managed by a multidisciplinary team. Cesarean delivery, and in selected patients, assisted second stage of labor, has been recommended in patients with aortopathies (153). Women with normal aortic dimensions and no residual isolated CoA may undergo vaginal delivery safely. Finally, given that the risk of CHD in the offspring of patients with isolated CoA is approximately 4–6%, and is higher in the presence of a bicuspid aortic valve (154), fetal echo has been recommended for all of these pregnancies as part of routine prenatal care (42).

## Conclusions

**Figure 7:**
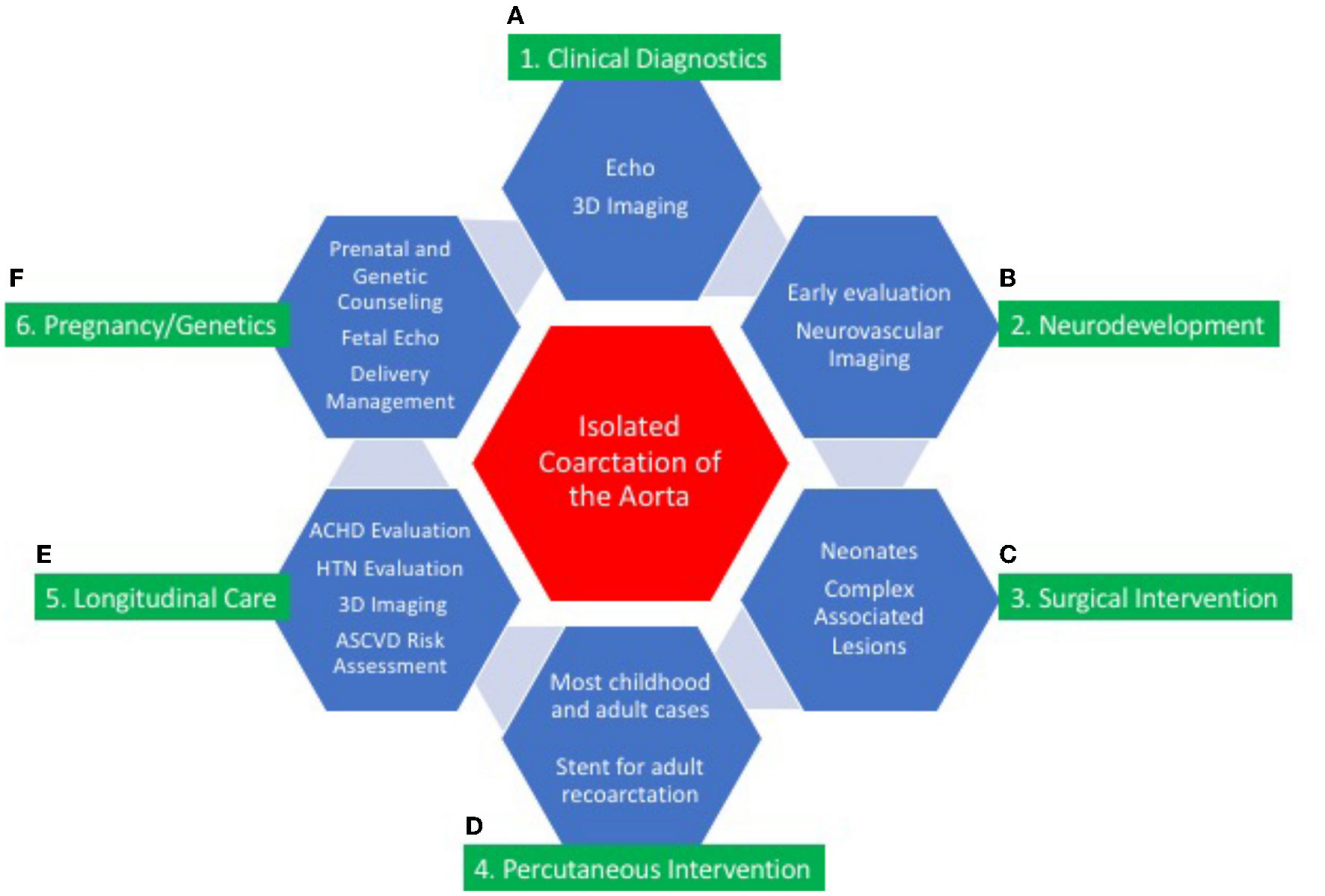
Central illustration: continuum of care for isolated coarctation of the aorta. The continuum of care for isolated coarctation of the aorta includes clinical diagnostics **(A)**; neurodevelopmental assessment **(B)**; surgical **(C)** or percutaneous intervention **(D)**; longitudinal care during adulthood **(E)**; and pregnancy management including genetic counseling **(F)**. Individualized management strategies are determined by age at presentation, anatomic features of CoA, and associated conditions including other CHD and genetic syndromes.

Isolated CoA is a disease in which progressive pathophysiologic changes influence long-term outcome (Figure 7). The diagnosis of isolated CoA occurs most commonly during early childhood, can present later in life, and has excellent short and long-term outcomes. Fetal detection, prospective management of delivery in high-risk maternal cases, and genetic testing is recommended in consultation with multidisciplinary CV genetics services. End-to-end or extended end-to-end anastomosis is the favored surgical approaches for isolated CoA. Percutaneous transcatheter intervention in children and adults has proven to be successful in treating CoA. Primary stent placement is the procedure of choice in larger children and adults. Neurocognitive assessment is an essential component of multidisciplinary care.

Adults with treated isolated CoA can thrive with activities of daily living and childbirth with few limitations. Active surveillance in collaboration with an adult CHD program is recommended. Advances in imaging enable identification of aortic complications and myocardial dysfunction and guide individualized medical therapies and the need for re-intervention.

The management of isolated CoA is a success story, with improved prenatal diagnosis, high likelihood of long-term survival, and a better understanding of complications that may arise during adolescence and adulthood. Additional follow-up data continue to be needed to determine the severity and frequency of aortic aneurysm formation, myocardial dysfunction, the need for re-intervention, and whether lifestyle modifications in childhood may reduce late-onset atherosclerotic risks. All isolated CoA patients, whether repaired or not, benefit from lifelong CV care by a cardiologist familiar with this disease.

## Data Availability Statement

The original contributions presented in the study are included in the article/supplementary material, further inquiries can be directed to the corresponding author.

## Author Contributions

AB, ML-H, JV, and BK were responsible for final review submission. All authors contributed to the article, including editing, and approved the submitted version.

## Funding

Funding was provided *via* Institutional support to the co-authors who generated this review.

## Conflict of Interest

The authors declare that the research was conducted in the absence of any commercial or financial relationships that could be construed as a potential conflict of interest.

## Publisher's Note

All claims expressed in this article are solely those of the authors and do not necessarily represent those of their affiliated organizations, or those of the publisher, the editors and the reviewers. Any product that may be evaluated in this article, or claim that may be made by its manufacturer, is not guaranteed or endorsed by the publisher.

## Abbreviations

CPB: cardiopulmonary bypass
CHD: congenital heart disease
isolated CoA: coarctation of the aorta
Echo: echocardiography
HTN: hypertension
PDA: patent ductus arteriosus
SI: stent implantation
TS: Turner Syndrome.
